# Inhibition of glucosylceramide synthase eliminates the oncogenic function of p53 R273H mutant in the epithelial-mesenchymal transition and induced pluripotency of colon cancer cells

**DOI:** 10.18632/oncotarget.11169

**Published:** 2016-08-10

**Authors:** Salman B. Hosain, Sachin K. Khiste, Mohammad B. Uddin, Vindya Vorubindi, Catherine Ingram, Sifang Zhang, Ronald A. Hill, Xin Gu, Yong-Yu Liu

**Affiliations:** ^1^ Department of Basic Pharmaceutical Sciences, University of Louisiana at Monroe, Monroe, LA 71201, USA; ^2^ Department of Integrated Chinese and Western Medicine, The Second Xiangya Hospital of Central South University, Changsha, Hunan 410011, China; ^3^ Department of Pathology, Louisiana State University Health Sciences Center, Shreveport, LA 71130, USA

**Keywords:** tumor suppressor p53, epithelial-mesenchymal transition, cancer stem cells, glucosylceramide synthase, missense mutation

## Abstract

Missense mutation of tumor suppressor p53, which exhibits oncogenic gain-of-function (GOF), not only promotes tumor progression, but also diminishes therapeutic efficacies of cancer treatments. However, it remains unclear how a p53 missense mutant contributes to induced pluripotency of cancer stem cells (CSCs) in tumors exposed to chemotherapeutic agents. More importantly, it may be possible to abrogate the GOF by restoring wild-type p53 activity, thereby overcoming the deleterious effects resulting from heterotetramer formation, which often compromises the efficacies of current approaches being used to reactivate p53 function. Herewith, we report that p53 R273H missense mutant urges cancer cells to spawn CSCs. SW48/TP53 cells, which heterozygously carry the p53 R273H hot-spot mutant (R273H/^+^, introduced by a CRISPR/Casp9 system), were subchronically exposed to doxorubicin in cell culture and in tumor-bearing mice. We found that p53-R273H (TP53-Dox) cells were drug-resistant and exhibited epithelial-mesenchymal transition (EMT) and increased numbers of CSCs (CD44v6^+^/CD133^+^), which resulted in enhanced wound healing and tumor formation. Inhibition of glucosylceramide synthase with *d-threo*-1-phenyl-2-decanoylamino-3-morpholino-1-propanol (PDMP) sensitized p53-R273H cancer cells and tumor xenografts to doxorubicin treatments. Intriguingly, PDMP treatments restored wild-type p53 expression in heterozygous R273H mutant cells and in tumors, decreasing CSCs and sensitizing cells and tumors to treatments. This study demonstrated that p53-R273H promotes EMT and induced pluripotency of CSCs in cancer cells exposed to doxorubicin, mainly through Zeb1 and β-catenin transcription factors. Our results further indicate that restoration of p53 through inhibition of ceramide glycosylation might be an effective treatment approach for targeting cancers heterozygously harboring TP53 missense mutations.

## INTRODUCTION

The p53 protein, encoded by human gene *TP53,* functions as a key tumor suppressor that stabilizes the genome with respect to propensity for tumorigenesis and cancer progression. The *TP53* gene is somatically mutated in over half of all cancer cases. More than 80% of *TP53* alterations are missense mutations, encoding full-length and dysfunctional proteins [1, 2]. Alterations at codons 175, 248, and 273 constitute 19% of all *TP53* mutations reported, and are considered to be mutation hotspots in human cancers, including those occurring in colon and lungs [1–3] (http://p53.free.fr/Database/p53_cancer/all_cancer.html). Missense versions of p53 that lack the tumor suppression activity of wild-type p53 (wt p53) instead often exhibit oncogenic gain-of-function (GOF) [4]. Knock-in mouse models that express hotspot mutant alleles R172H or R270H (R175H or R273H in the human versions) manifest GOF by conferring a broader tumor spectrum and more tumor metastases, as compared with wt p53-expressing mice [5, 6]. *TP53* mutants are observed more frequently in tumors diagnosed at advanced stages, or with more metastases, and in recurrences of cancer in colon, ovaries and breasts [7–9].

Despite the well-known fact that expression of p53 mutants correlates strongly to poor prognosis in cancer patients, the exact roles in the promotion of cancer progression played by p53 mutants, which vary in type as well as position, remain as yet unclear. Recent reports document that inactivation of p53 function enhances the production efficiency, and decreases the latency for emergence of induced pluripotent stem cells (iPSCs) in cell culture [10, 11]. iPSCs can be generated from somatic cells of mouse and of human by introduction of Oct4, Sox2, Klf4 and c-Myc transcription factors [12]. Suppression of p53 with small interfering RNA (siRNA) increased the efficiency of iPSC generation from human fibroblasts, indicating that the p53-p21 pathway serves as a barrier to iPSC generation [13]. With Oct4 and Sox2 reprogramming, p53-knockout cells merely maintained their pluripotent capacity *in vivo*, whereas mutant p53 (R172H) mouse cells gave rise to malignant tumors, with inherent oncogenic GOF attributable to the involvement of Klf4 [14].

Metastasis and drug resistance are major limitations to the survival and management of patients with cancers. Cancer stem cells (CSCs), which possess malignant capacities of self-renewal and pluripotency, not only lead tumorigenesis, but also drive tumor progression and are responsible for treatment failure [15, 16]. Most cytotoxic agents in chemotherapy damage DNA or disrupt mitosis to induce cell death in highly proliferative populations of cancer cells. Chemotherapy often eliminates differentiated cells in tumors, but surviving CSCs (which do not rapidly proliferate) may cause disseminated metastases or recurrence of aggressive tumors upon treatment failure. Recent studies have convincingly shown enrichment with CSCs in populations of breast, ovarian and colon cancer cells that have become drug resistant following sequential exposure to anticancer drugs, including doxorubicin, paclitaxel and 5-fluorouracil (5FU) [17–19]. Enrichment with CSCs is also observed in xenogeneic tumors following chemotherapies [20, 21]. Similarly, after systemic chemotherapy of cancer patients, CSCs are increased in breast and lung cancers [22, 23].

Aiming to understand the roles played by p53 mutants in promoting tumor progression and in chemotherapy failure, we studied EMT and CSC populations pursuant to various treatments in cancer cells heterozygously carrying a p53 missense mutation, in cell culture and in tumor-bearing mice.

## RESULTS

### R273H p53 mutant is a promoting factor for drug resistance and induced EMT of colon cancer cells

**Figure 1:**
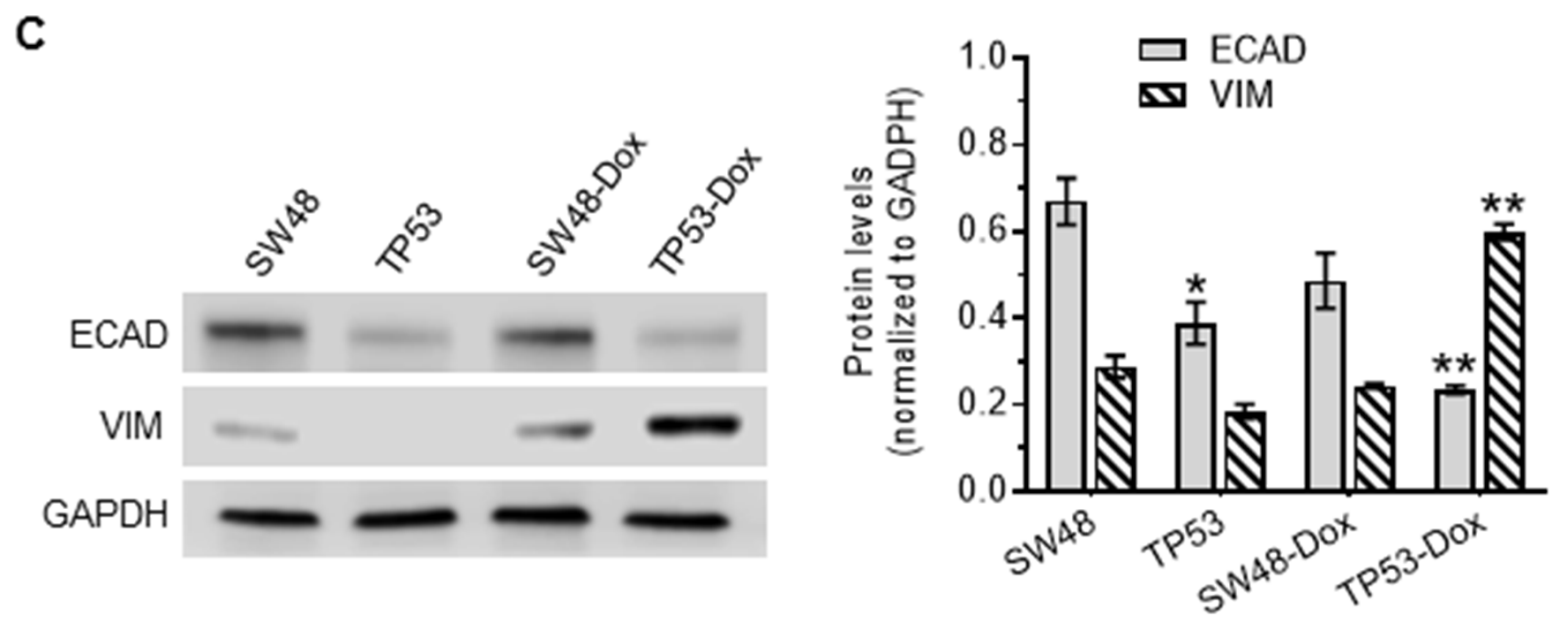
p53 missense mutation and cancer cell response to doxorubicin Cells were treated with doxorubicin in 5% FBS medium for 72 hr. **A.** Cell response to doxorubicin. MCF-12A (wt p53), SW48 (wt p53), COLO 320DM (mutant p53 R248W; COLO), WiDr (mutant p53 R273H), SW48/TP53 (mutant p53 R273H), SW48-Dox and TP53-Dox (mutant p53 R273H) cells were treated with doxorubicin for 72 hr. *, *p*<0.001, compared to wt p53 cells (MCF-12A, SW48). **B.** Doxorubicin induced EMT in cancer cells carrying a p53 R273H mutant allele. Cells (passages 8-10) of SW48, SW48/TP53, SW48-Dox and TP53-Dox were grown in multi-chamber slides with 10% FBS medium for 48 hr. Red, Alexa Fluor 555–vimentin (VIM); green, Alexa Fluor 488–E-cadherin (ECAD); blue, DAPI–nucleus. Scale bar represents 50 μm in photomicrographs (200x magnification). **C.** Western blotting for EMT marker assessment. Equal amounts of extracted detergent-soluble proteins (50 μg protein/lane) were resolved using 4-20% gradient SDS-PAGE and then immunoblotted with antibodies of E-cadherin (ECAD), vimentin (VIM) or GAPDH. Protein levels were represented by ratios (mean ± SD) of ECAD or VIM densities normalized against GAPDH from three blots. *, *p*<0.01 compared to SW48 cells; **, *p*<0.001 compared to SW48-Dox cells.

It has been reported that p53 missense mutants, including those at “hotspot” codons 248 and 273, conferred oncogenic GOF in cells and in transgenic mice [24, 25]. We examined cell response to doxorubicin in human COLO 320DM (COLO) and WiDr colon cancer cells, which carry homozygous p53-R248W and p53-R273H mutations [26, 27], respectively. COLO and WiDr cells were significantly resistant to doxorubicin as compared to wt p53 SW48 colon cancer cells, or MCF-12A noncancerous epithelial cells (Figure 1A left-panel). The IC_50_ values for doxorubicin in COLO and WiDr are 36-fold (1.50 *vs.* 0.04 μM, p<0.001) and 18-fold (0.78 vs. 0.04, p<0.001) higher than in SW48 cells. Other missense mutant SW48/TP53 (TP53) cells, which heterozygously carry p53-R273H knocked in by using a CRISPR/Cas9 genome editing system [28], however, showed responses to doxorubicin similar to those of its parental SW48 colon cancer line (wt p53) (Figure 1A right-panel). To characterize the association of GOF with acquired drug resistance during chemotherapy, we cultured TP53 as well as SW48 cells in 10% FBS medium with sub-lethal concentrations of doxorubicin (5-25 nM) for approximately 26 passages. As shown in Figure 1A (right-panel), exposure to doxorubicin induced drug resistance in heterozygous p53-R273H mutant cells. The IC_50_ value for doxorubicin in TP53-Dox cells increased by 24-fold (1255 *vs.* 49.2 nM, p<0.001) over that seen for naïve SW48/TP53 cells; however, the IC_50_ values in SW48-Dox cells did not change significantly (45 *vs.* 50 nM) versus naïve SW48 cells (Figure 1A right panel).

GOF associated with the homozygous presence of mutant proteins (R273H, R175H) has been reported to induce EMT in colon cancer WiDr, endometrial cancer HEC-50, and prostate cancer DU145 cells [29–31]. Until now, the question as to whether or not the heterozygous presence of a particular missense p53 mutation might play any significant role(s) in the emergence of EMT during cancer progression had remained unanswered. In our EMT study, we did not find obvious alterations in morphology, or in EMT-hallmark E-cadherin (ECAD) or vimentin (VIM) expression changes, in TP53 cells with heterozygous p53-R273H, as compared to SW48 cells (Figure 1B left-panels). Interestingly, doxorubicin exposure induced a shift in cell morphology from a paved stone epithelial appearance (in TP53 cells) to phenotypes of a more mesenchymal character in TP53-Dox cells, with loss of cell-to-cell contact and increased cell spreading (Figure 1B right-panels). These morphological changes were accompanied by the upregulated expression of mesenchymal marker VIM, and decreased expression of the epithelial marker ECAD (Figure 1B, 1C). These results indicate that GOF associated with the heterozygous presence of p53-R273H promotes drug resistance and EMT in cancer cells exposed to anticancer drugs.

### p53-R273H mutant promotes the pluripotency of colon cancer cells exposed to doxorubicin

**Figure 2:**
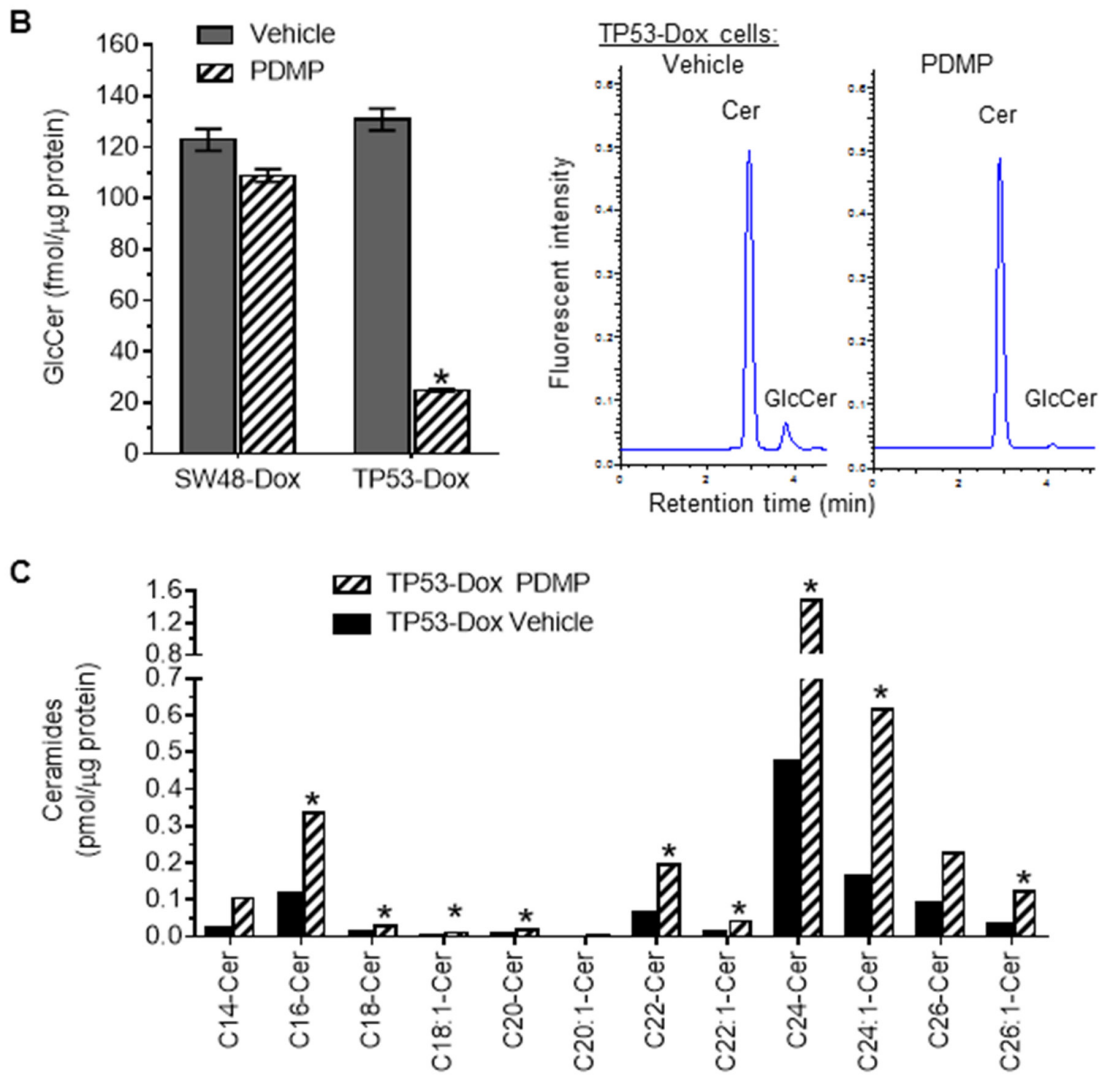
Effect of p53 R273H mutant on wound healing of colon cancer cells **A.** Wound healing of cancer cells. Scale bar represents 50 μm in photomicrographs (×100 magnification). *, *p*<0.001 compared to TP53 cells; **, *p*<0.001 compared to SW48-Dox or TP53-Dox cells treated with vehicle. **B.** GCS activities analyzed by HPLC. Cer, NBD C6-ceramide. GlcCer, NBD C6-glucosylceramide. *, *p*<0.001 compared to Vehicle of TP53-Dox cells. **C.** Ceramides analyzed by ESI/MS/MS. Cer, ceramide. *, ≥twofold higher than vehicle.

We carried out experiments with a wound healing assay and observed a significant increase in migration ability of TP53-Dox cells, as compared with TP53 cells or SW48-Dox cells (Figure 2A). The wound healing seen with TP53-Dox cells was increased by more than twofold (83% *vs.* 29.9%, p<0.001) as compared to the Dox-naïve TP53 cells, and was also significantly higher than for SW48-Dox cells (Figure 2A). In contrast, the wound healing was not significantly different between SW48-Dox and SW48 cells. Furthermore, we treated SW48-Dox and TP53-Dox cells with PDMP, a glucosylceramide synthase (GCS) inhibitor [32, 33]. Interestingly, we found that PDMP treatments significantly reduced wound healing of TP53-Dox cells, by more than twofold (36 *vs*. 83%, p<0.001; Figure 2A). PDMP treatments reduced GCS activities in TP53-Dox cells, by more than fivefold (25 *vs.* 131 fmol/μg protein, p<0.001), but not in SW48-Dox cells (Figure 2B). PDMP treatments doubled cellular levels of several species of ceramides (Cers), including C14-Cer, C18-Cer, C20-Cer, C22-Cer, C24:1-Cer and C26:1-Cer in TP53-Dox cells, as detected by ESI/MS/MS analysis (Figure 2C).

**Figure 3:**
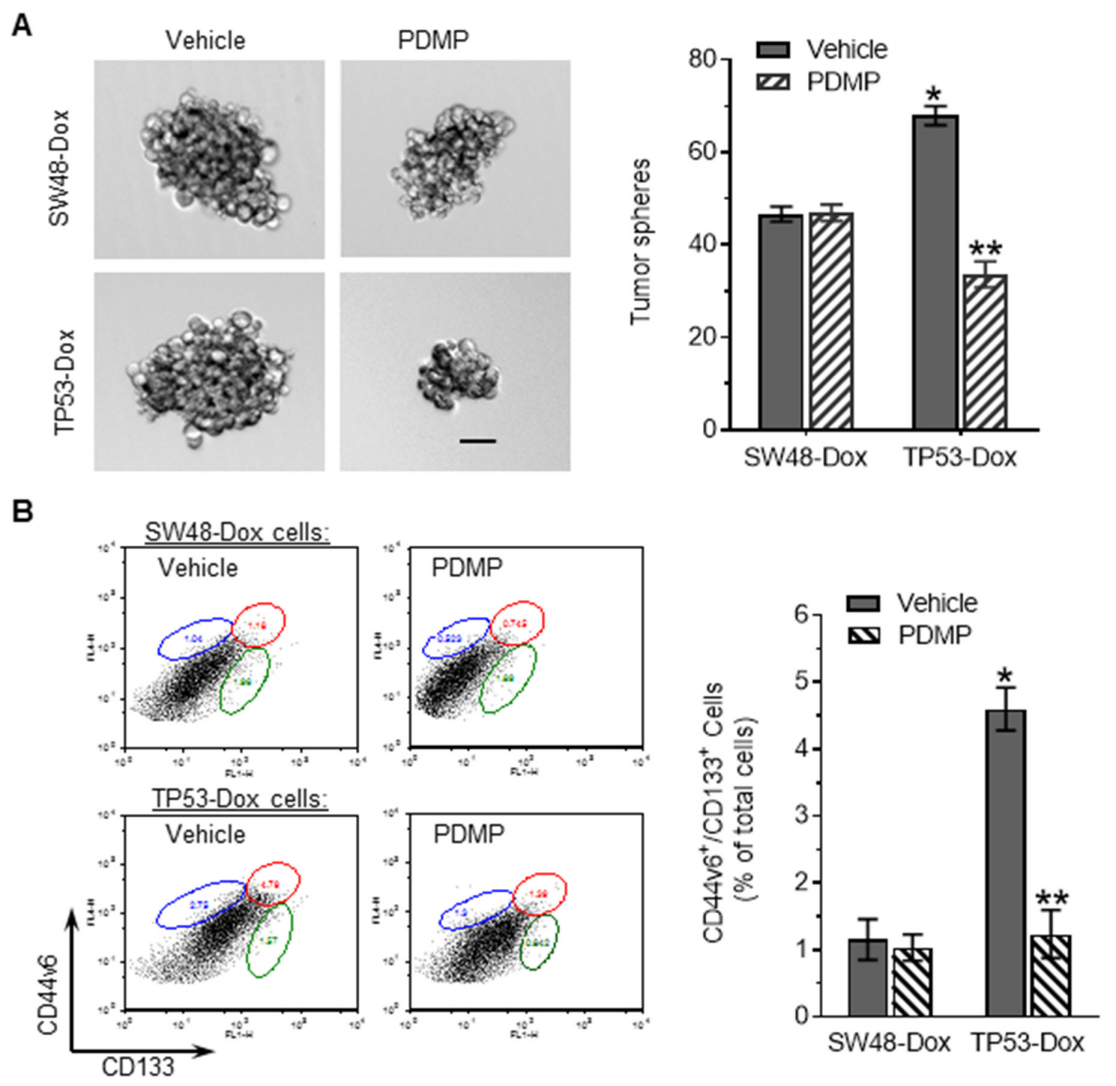
p53 R273H mutant enhanced tumor sphere formation and CSCs among colon cancer cells Cells of SW48-Dox and TP53-Dox lines were separately treated with 5 μM PDMP in 5% FBS medium. **A.** Tumor sphere formation. Scale bar represents 50 μm in photomicrographs (100x magnification). *, *p*>0.001 compared to SW48-Dox with vehicle; **, p<0.001 compared to TP53-Dox with vehicle. **B.** CSCs. Cells were incubated with Alexa-Fluor488 CD44v6 and APC-CD133 antibodies and analyzed by using flow cytometry. The detected CD44v6^+^/CD133^+^ cells (CSCs) are identified in the plots by enclosure with an ellipse (upper right), and compared with vehicle controls, as percentages of total cells in the adjacent bar graph.

In addition to drug resistance, EMT plays critical roles in modulating cancer stem-like cell phenotype [34]. To test possible roles played by p53 R273H mutant in acquisition of cancerous stemness, we further investigated the effects of R273H on the self-renewal potential of cells using a sphere formation assay. We found that the numbers of enlarged tumor spheres increased in TP53-Dox cells, comparing to SW48-Dox cells. PDMP treatments substantially decreased sphere numbers, by twofold (33 *vs.* 67, p<0.001) in TP53-Dox cells (Figure 3A); however, PDMP treatment did not significantly alter tumor spheres in SW48-Dox cells. We next looked for CSCs with CD44v6^+^/CD133^+^ phenotype among cells in these lines, as a CD44v6^+^/CD133^+^ phenotype has been associated with CSCs in tumors and in cells of colon cancers [35]. It was found that CSC populations increased fourfold in TP53-Dox cells (1.2 *vs.* 4.6%, p<0.001) as compared to in SW48-Dox cells (Figure 3B). With PDMP treatment, CSC populations in TP53-Dox cells significantly decreased, by approximately 3.5-fold (4.6% *vs.* 1.23%, p<0.001), whereas PDMP treatments did not have a significant effect in SW48-Dox cells (Figure 3B).

### p53 R273H mutant promotes the induced pluripotency of cancer cells through Zeb1 and c-Myc transcription factors

**Figure 4:**
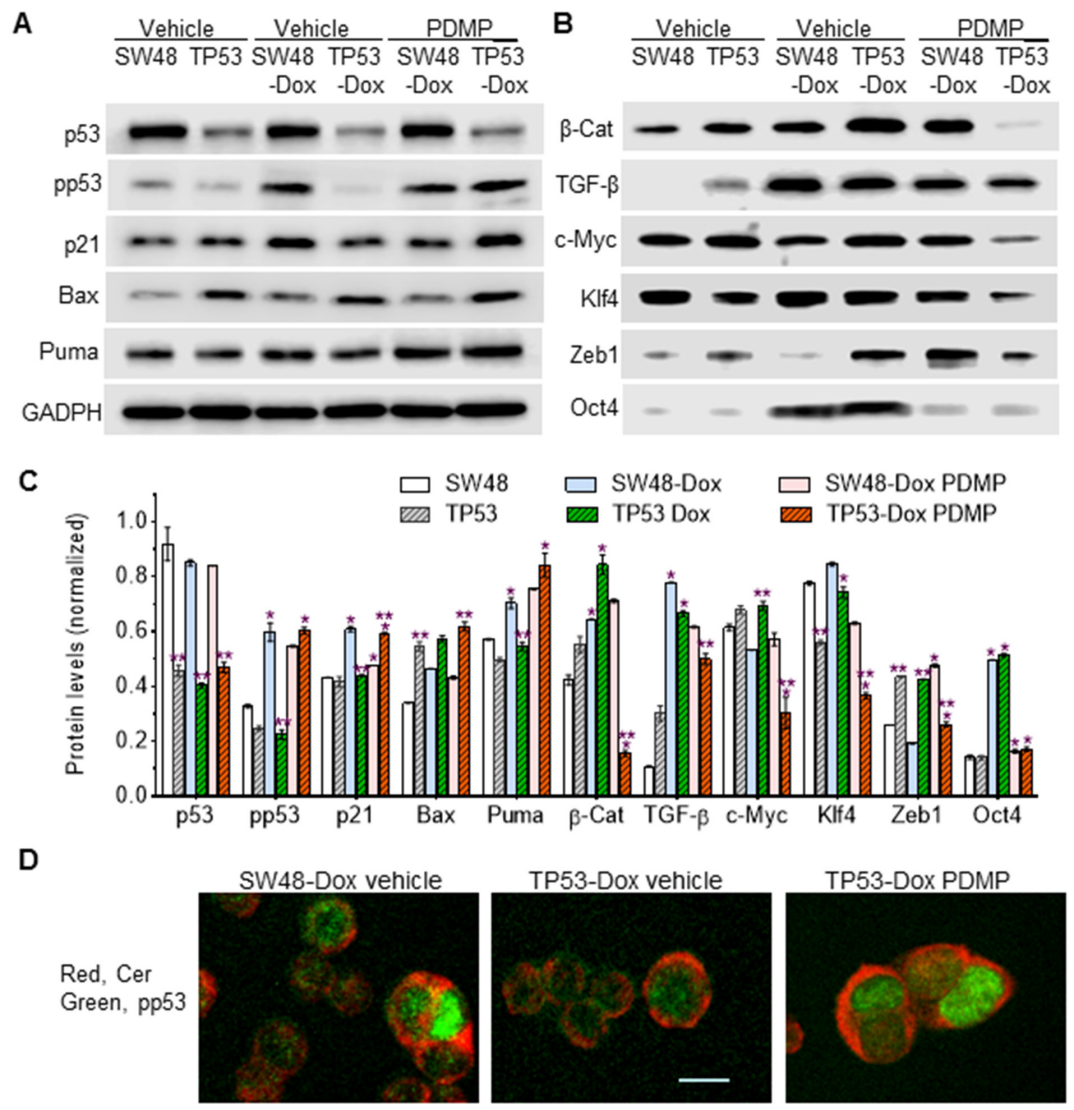
p53 expression and p53-responsive proteins in R273H p53 mutant cells Cells were treated with PDMP (5 μM, 6 days) followed by doxorubicin exposure (50 nM, 48 hr). Equal amounts of detergent-soluble proteins (50 μg/lane) were resolved by 4-20% SDS-PAGE and immunoblotted with corresponding primary and secondary antibodies, sequentially. **A.** Effects of doxorubicin-induction and PDMP on p53 and p53-responsive protein expression. **B.** p53 modulates iPSC factor expression in cancer cells exposed to Dox. PDMP inhibits ceramide glycosylation, thereby increasing cellular ceramide levels, which may favor wt p53 expression over that of the R273H missense mutant. **C.** Protein expression levels. Protein levels are presented here as ratios of their densities normalized against GAPDH from three Western blots. *, p<0.05 compared to parental (SW48, TP53) or Dox-induced sublines (SW48-Dox, TP53-Dox); **, p<0.05 compared to SW-48, or SW48-Dox and SW48-Dox PDMP. pp53, phosphorylated p53 (Ser15); β-Cat, β-catenin; TGF-β, transforming growth factor β. **D.** Immunostaining of ceramide and pp53. The scale bar represents 5 μm in photomicrographs (200x magnification).

To understand how R273H impacts pluripotency of cancer cells, we assessed the protein expression of p53 and p53-responsive genes. A previous report showed that isogenic TP53 cells expressed the R273H mutant in response to a presence of 5-fluorouracil [28]. In the present study, p53 expression, in TP53 and TP53-Dox cells, in each case assessed either as pan-p53 or as phosphorylated p53 only (pp53, Ser15), was not altered in response to DNA damage induced by doxorubicin, in contrast to behavior of SW48 and SW48-Dox cells, which carry wt p53 (Figure 4A, 4C). Significantly, the protein levels of p53, pp53, p21 and Puma were decreased in TP53-Dox cells as compared to SW48-Dox cells. Long-term Dox exposure (26-passages) significantly enhanced the protein levels of β-catenin, TGF-β, and Oct4 in both SW48-Dox and TP53-Dox cell sublines, as compared to naïve SW-48 and TP53 cells, respectively (Figure 4B, 4C). However, Dox exposure increased c-Myc and Zeb1 levels exclusively in TP53-Dox, but not in SW48-Dox cells (Figure 4B, 4C).

Our previous studies showed that ceramide can restore wt p53 expression and functional activity in ovarian cancer OVCAR-8 and NCI/ADR-RES cells that carry p53 deletion mutations in codons 126-132 and 126-133, respectively [32, 33]. Until now, it remained unknown whether or not cells harboring p53 missense mutants could be restored to express wt p53 protein. Intriguingly, we found that PDMP treatments, which inhibited GCS and increased cellular ceramide (Figure 2B, 2C, 4D), restored wt p53 expression as well as p53-responsive proteins in TP53-Dox cells (Figure 4A, 4B, 4C, 4D). The levels of pp53, and of several proteins encoded by p53-responsive genes (p21, Bax and Puma), are substantially enhanced in TP53-Dox cells treated with PDMP, and those levels are almost equal to those observed in SW48 cells that carry wt p53 (Figure 4A). Moreover, PDMP treatments exclusively decreased the protein levels of β-catenin, TGF-β, c-Myc, Klf4, and Zeb1 in TP53-Dox cells (Figure 4B, 4C), whereas PDMP treatment significantly lowered Oct4 in both SW48-Dox and TP53-Dox cells. These results, taken in aggregate with the observed array of expression level changes between cell lines and sub-lethal Dox exposure, indicate that increased ceramide levels brought about by inhibition of GCS restores wt p53 expression in cells harboring a p53-R273H mutant allele. Also, the promoting effects of p53 R273H mutation on the spawning of induced cancer stem cells are seen to be highly associated with Zeb1, c-Myc, β-catenin, and Klf4.

### p53 R273H mutant promotes induced pluripotency of cancer stem cells in tumors during chronic sub-lethal doxorubicin treatments

**Figure 5:**
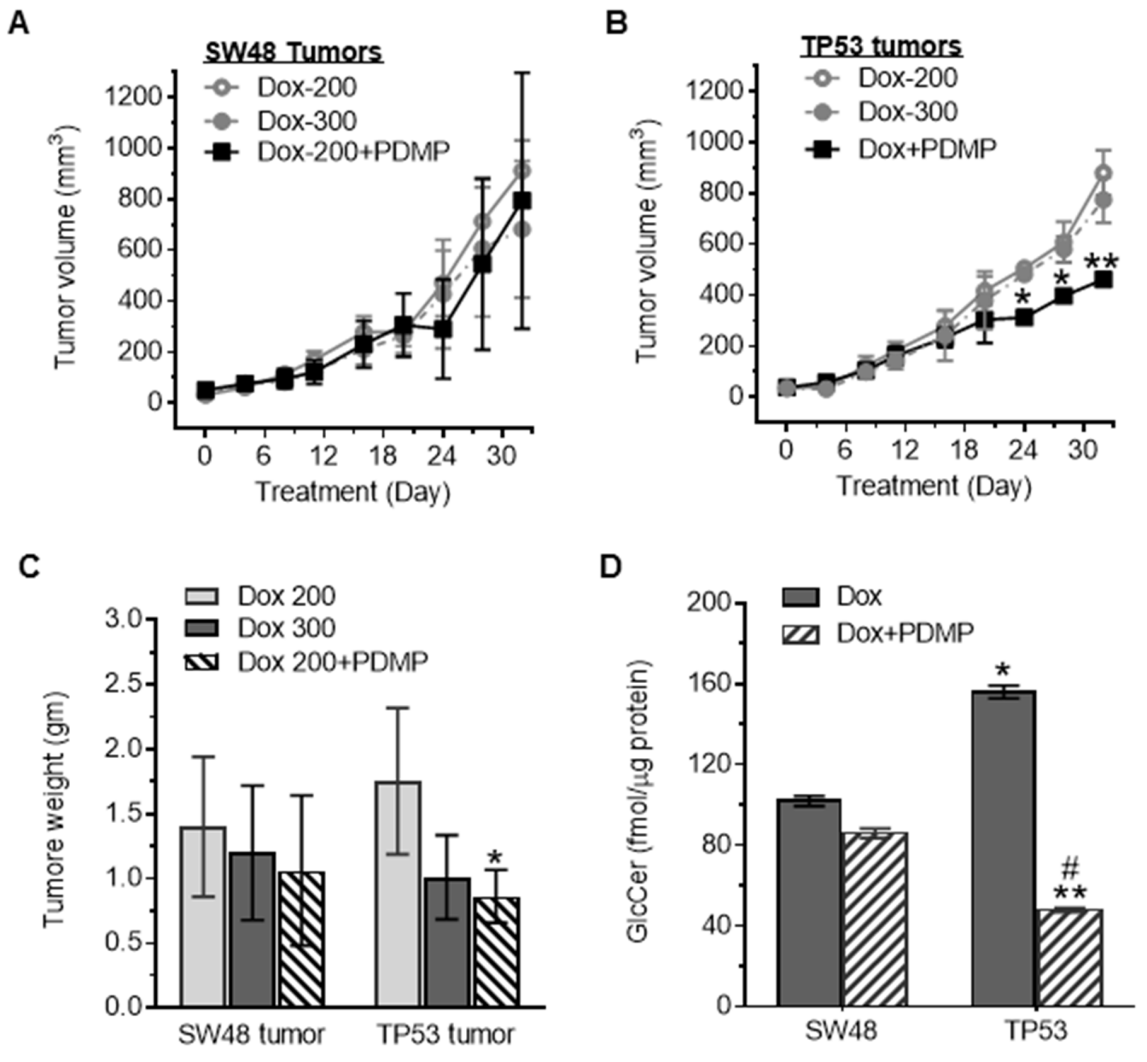
Tumor responses to doxorubicin treatments Cells of SW48 and SW48/TP53 (TP53) lines were subcutaneously inoculated into athymic nude mice. Low doses of doxorubicin (200 μg/kg and 300 μg/kg, per 6 days, i.p.) and PDMP (4.0 mg/kg, per 3 days, i.p.) were administered for 32 days (5 cases/group). **A.** SW48 tumor growth. **B.** TP53 tumor growth. *, *p*<0.01, compared to doxorubicin alone treatments. **C.** Tumor weight after treatments. *, *p*<0.01, compared to doxorubicin alone treatments. **D.** GCS activities in tumors after treatments. *, p<0.001, compared to SW48 tumors treated with Dox (200 μg/kg); **, *p*<0.001, compared to TP53 tumors treated with doxorubicin (200 μg/kg); #, p<0.001, compared to SW48 tumors treated with Dox and PDMP.

To further examine whether p53-R273H promotes iPSCs in tumors, we treated mice bearing SW48 and TP53 (R273/+) tumor xenografts with lower (sub-therapeutic) doses of doxorubicin. We did not observe any significant alteration of tumor growth between TP53 and SW48 tumor-bearing mice after doxorubicin treatments (200, 300 μg/kg) (Figure 5A-5C). However, PDMP treatments (4.0 mg/kg) significantly decreased tumor growth and tumor weight in TP53 tumor-bearing mice (Figure 5A-5C), but not in those with tumors generated from SW48 cells. HPLC analysis indicated that GCS activities in TP53-generated tumors were significantly higher (156 *vs.* 102 fmol/μg, p<0.001) than in SW48 tumors of mice treated with sub-therapeutic doses of doxorubicin (200 μg/kg) (Figure 5D). PDMP treatments significantly decreased GCS activities, more than threefold (156 *vs.* 48 fmol/μg, p<0.001) versus those seen in TP53 tumors treated with doxorubicin alone (Figure 5D).

**Figure 6:**
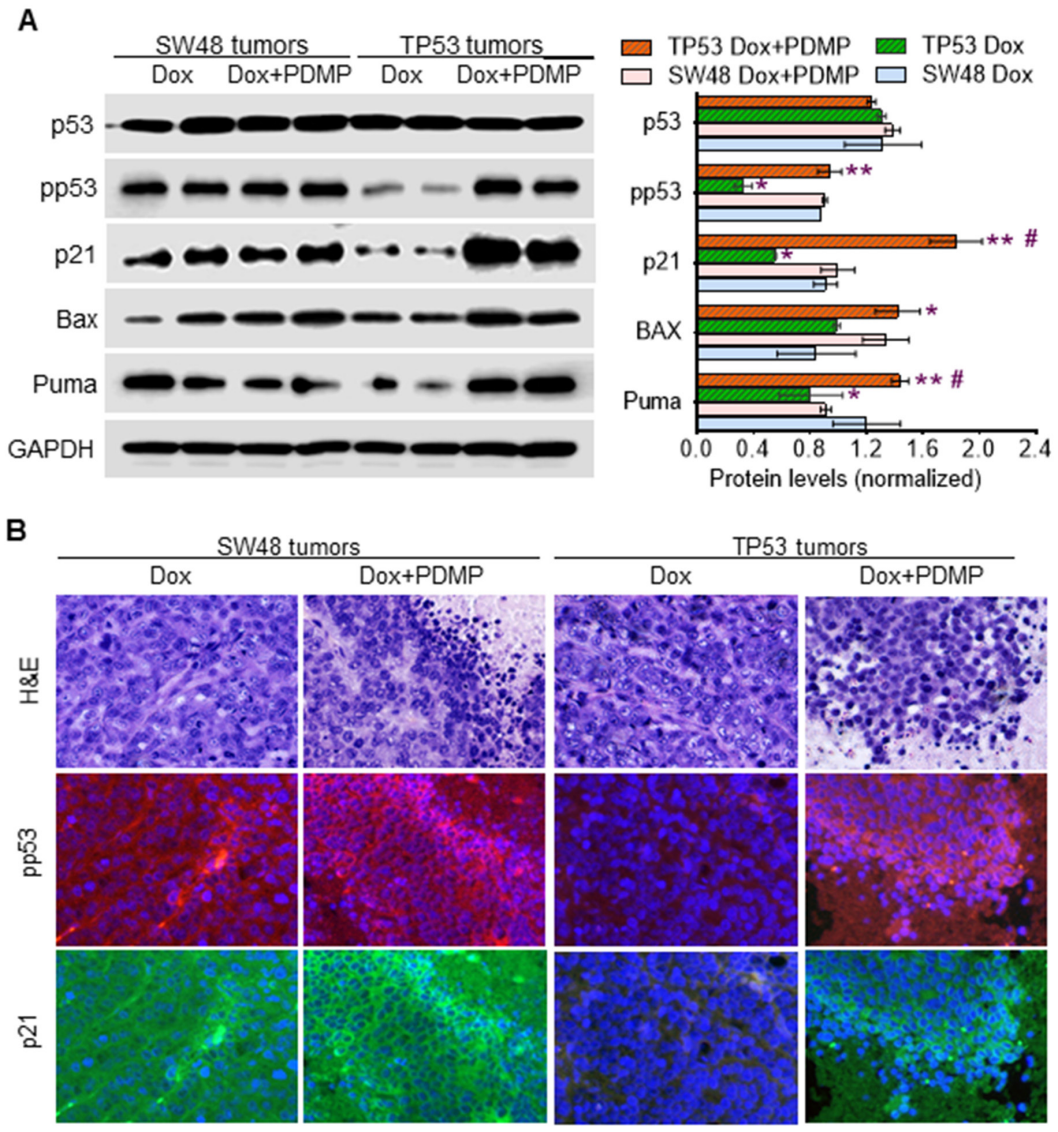
Inhibition of GCS restored p53 expression in tumors of mice during doxorubicin treatments **A.** Western blotting. Equal amounts of detergent-soluble proteins (50 μg/lane) extracted were resolved using 4-20% gradient SDS-PAGE and then immunoblotted with corresponding antibodies. Protein levels in the bar graph are presented as mean±SD of their densities normalized against GAPDH from three blots. *, *p*<0.01 compared to SW48 tumors treated with Dox; **, *p*<0.01 compared to TP53 tumors treated with Dox; #, p<0.01 compared to SW48 tumors treated with Dox and PDMP. **B.** Immunostaining of pp53 and p21. The scale bar indicates 25 μm in photomicrographs (200x magnification). Red, Alexa Fluor 555-pp53; green, Alexa Fluor 488-p21; blue, DAPI-nucleus. The scale bar indicates 50 μm in photomicrographs (200x magnification).

Western blot analysis of TP53-generated tumors showed a pronounced dearth of pp53 (∼3-fold less), and of the protein products of the p53-responsive genes, including p21, Bax and Puma, as compared to levels seen for SW48-generated tumors (Figure 6A). PDMP treatments substantively restored wt p53 expression and downstream responses in tumors carrying p53 R273H mutation: the protein levels of pp53 (>3-fold), p21 (>3-fold), Bax and Puma are substantially increased in TP53 tumors treated with PDMP combined with doxorubicin, exhibiting levels almost equal to those seen in SW48-generated tumors, which carry wt p53 (Figure 6A). The immunohistochemistry findings were concordant, as we once again observed that combining PDMP with sub-chronic Dox treatment increased pp53 and p21 in TP53 tumors (Figure 6B).

**Figure 7:**
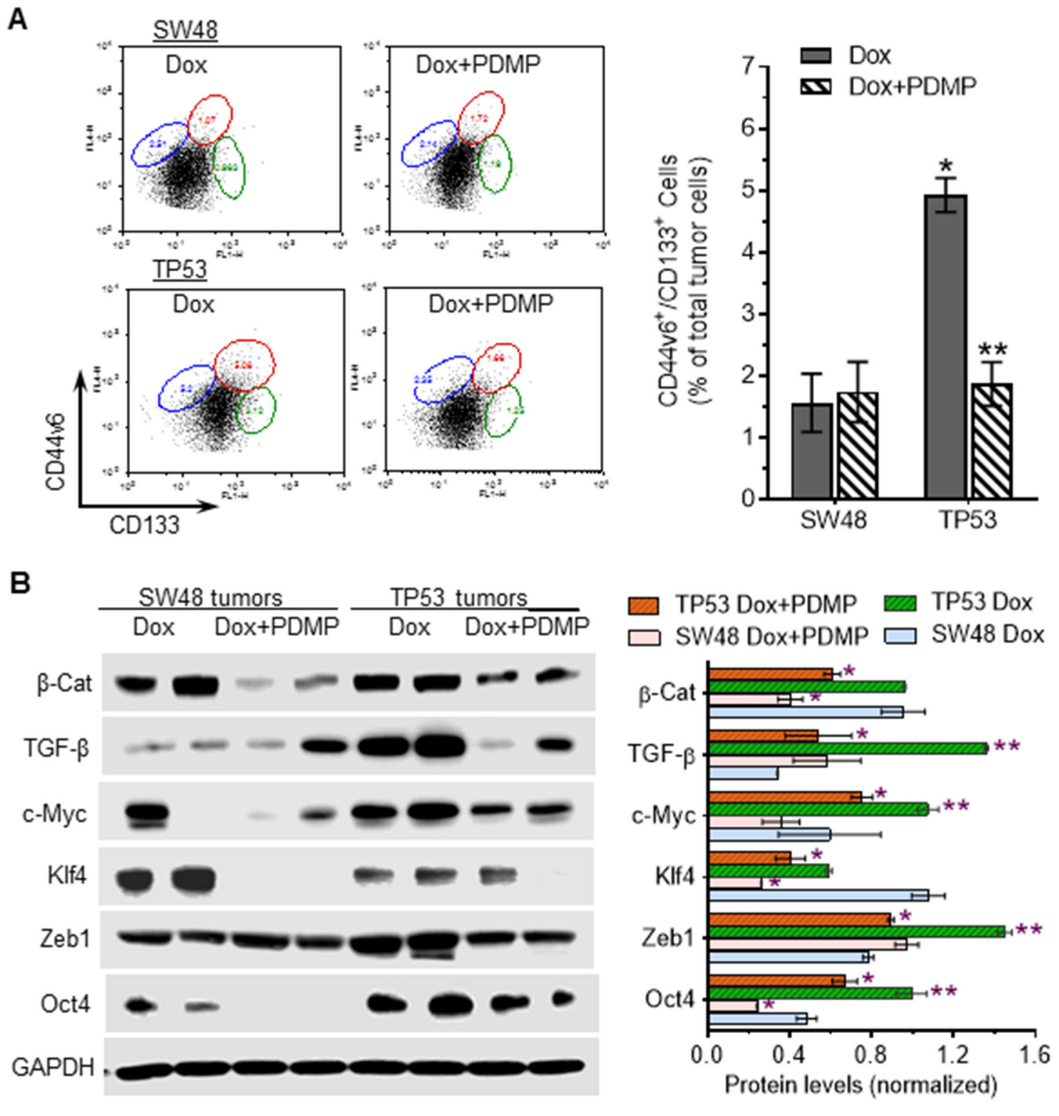
Effects of R273H p53 mutant on iPSC in tumors exposed to doxorubicin **A.** Flow cytometry analysis of colon CSCs (CD133+/CD44V6) from tumors in mice treated with doxorubicin (Dox, 200 μg/kg, per 6-days, i.p.) alone or combined with PDMP (4.0 mg/kg, per 3-days, i.p.), for 32 days. *, p<0.001 compared to SW48 tumors treated with Dox; **, p<0.001 compared to TP53 tumors treated with Dox. **B.** Western blotting of pluripotency regulators in tumors. Equal amounts of detergent-soluble proteins (50 μg/lane) extracted were resolved using 4-20% gradient SDS-PAGE and then immunoblotted with corresponding antibodies. Protein levels are represented as mean ± SD of their densities normalized against GAPDH from three blots. *, *p*<0.01 compared to Dox treatments (200 μg/kg); **, *p*<0.01 compared to SW48 tumors.

Furthermore, we found that sub-chronic doxorubicin treatments (200 μg/kg) significantly induced the numbers of CSCs (CD44v6^+^/CD133^+^), by threefold (4.9% *vs.* 1.7% of total tumor cells, *p*<0.001) in TP53 tumors, as compared to SW48-generated tumors in mice (Figure 7A). PDMP treatments dramatically decreased CSCs, by more than twofold (4.9% *vs*. 1.9% of total tumor cells, *p*<0.001) in TP53 tumors treated with PDMP combined doxorubicin, but not in SW48 tumors (Figure 7A). These PDMP-combined treatments did not significantly affect bone marrow stem cells (ABCG2^+^), nor did they impact GCS activities of bone marrow, either in SW48- or TP53-tumor-bearing mice (Supplementary Figure S1A, S1B).

Western blot analysis indicated that TGF-β, c-Myc, Zeb1 and Oct4 are pronouncedly increased in TP53-generated tumors after doxorubicin treatments, as compared to SW48-tumors (Figure 7B). PDMP treatments dramatically decreased the protein levels of β-catenin, c-Myc, Klf4, Zeb1 and Oct4 in TP53-tumors (Figure 7B), while these combined treatments significantly decreased only β-catenin, Klf4, and Oct4 in SW48-tumors. These findings further corroborate the results of cell-culture studies, demonstrating that GOF associated with p53 R273H mutation induces the production of iPSCs via Zeb1, c-Myc and TGF-β transcription factors.

### PDMP restores wild-type p53 expression in cells harboring p53 R273H mutant through ceramide

**Figure 8:**
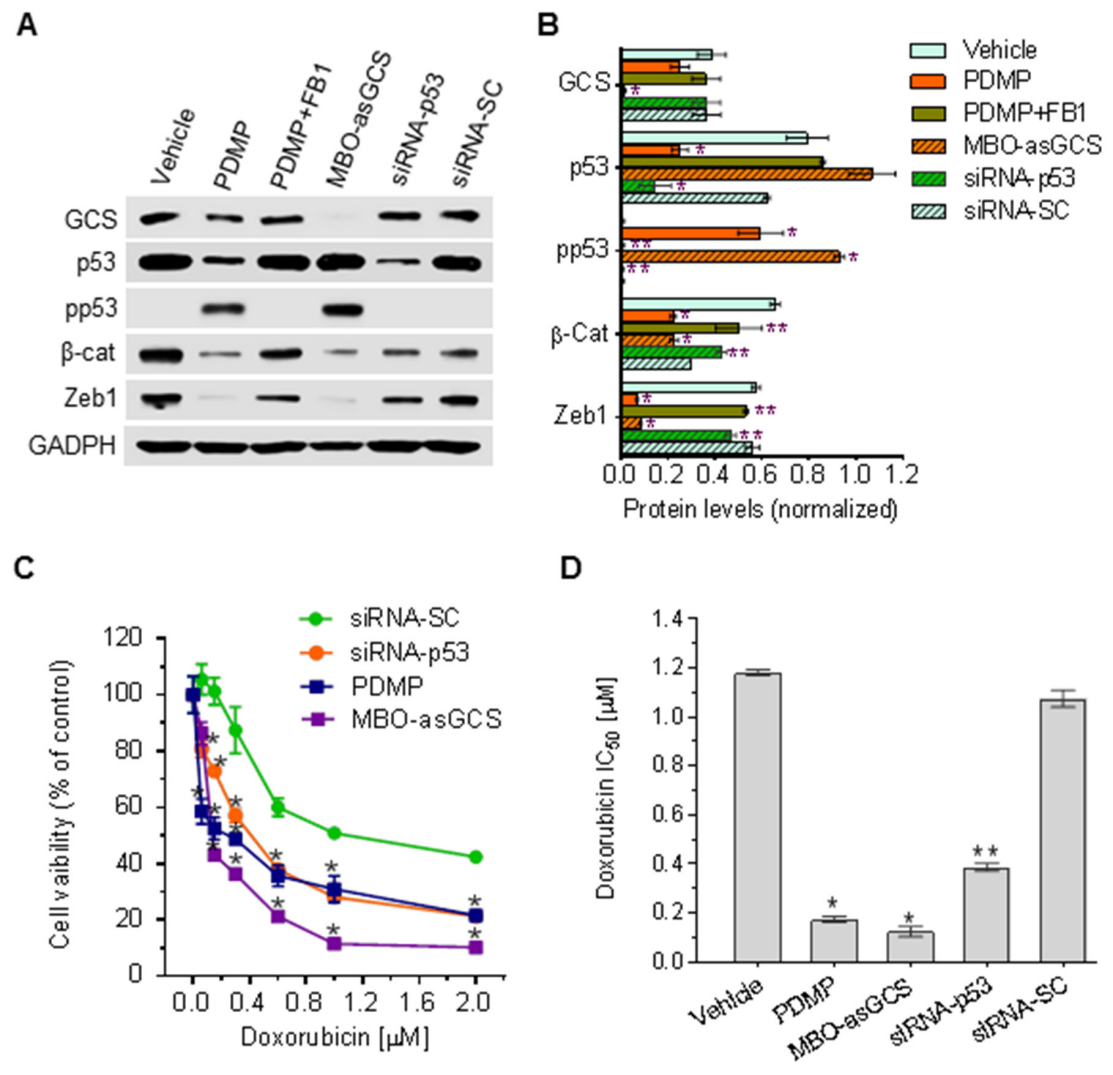
Ceramide restores wt p53 expression in cells carrying heterozygous R273H mutation After 48 h pretreatments with PDMP (5 μM), PDMP plus FB1 (25 μM), MBO-asGCS (100 nM), siRNA-p53 (100 nM), or siRNA-SC (100 nM), TP53-Dox cells were cultured in medium containing doxorubicin (50 nM) with each of these agents for 48 h to induce DNA damage and 72 h for cell viability assays. For the Western blot studies, the cells pretreated under each of the various sets of conditions, the doxorubicin (50 nM) exposure was combined with continuation of the various pretreatment agents for an additional 48 h to induce DNA damage before protein extraction. **A.** Western blotting. Equal amounts of detergent-soluble proteins extracted (50 μg/lane) were resolved using 4-20% gradient SDS-PAGE and then immunoblotted with corresponding antibodies; representative blots are presented. GCS, glucosylceramide synthase; pp53, phosphorylated p53 (Ser15); β-cat, β-catenin; siRNA-SC, siRNA scrambled control. **B.** Ceramide affects wt p53 expression. Protein levels are represented as mean ± SD of their densities normalized against GAPDH from three settings of blots. *, p<0.001 compared to vehicle or siRNA-SC; **, p<0.001 compared to PDMP or MBO-asGCS treatment. **C.** Cell responses to doxorubicin. **D.** IC_50_ values for doxorubicin. *, p<0.001 compared to siRNA-SC or vehicle. **, p<0.001 compared to PDMP or MBO-asGCS treatment.

**Figure 9:**
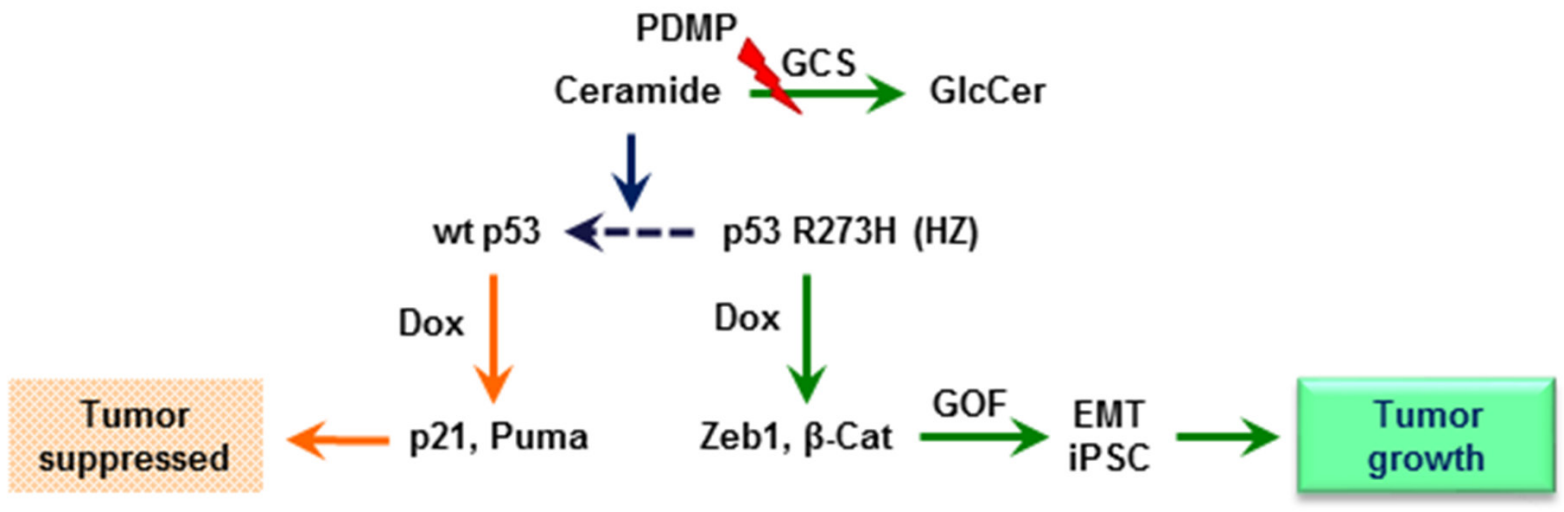
Ceramide restores wild-type p53 expression in cells heterozygously carrying a p53 R273H mutant allele When mutant-heterozygous (HZ) cancer cells are exposed to a sub-lethal dose of doxorubicin (Dox), DNA damage induces overexpression of p53 R273H, and the presence of this missense mutant protein upregulates Zeb1 and β-catenin (β-Cat) stem-like factors. The consequent gain-of-function (GOF) presents as epithelial-mesenchymal transition (EMT) and induced pluripotency of stem cells (iPSCs), lending to tumor growth. Inhibition of glucosylceramide synthase (GCS)-catalyzed ceramide glycosylation with PDMP increases cellular ceramide levels, bring about restoration of wt p53 protein expression, in turn increasing p53-responsive proteins, including p21 and Puma, thereby suppressing tumor progression.

GCS converts ceramide to glucosylceramide (GlcCer). This transformation strongly contributes to the regulation of cellular ceramide levels, as well as providing GlcCer as a precursor for biosynthetic elaboration to a number of other glycosphingolipids. Consistent with the above-described studies of PDMP as a means of substantively restoring wt p53, inhibition of ceramide glycosylation catalyzed by GCS, either by directly inhibiting GCS catalysis with PDMP (Figure 2B), or by silencing GCS expression with a mixed backbone oligonucleotide-antisense GCS (MBO-asGCS) [36], significantly increased levels of pp53 (>50-fold), decreased protein levels of β-catenin (3-fold) as well as of Zeb1 (8-fold) (Figure 8A, 8B), and accordingly sensitized TP53-Dox cells to doxorubicin, by approximately sevenfold (0.17 *vs.* 1.18 μM) and tenfold (0.12 *vs.* 1.18 μM), respectively (Figure 8C, 8D). However, fumonisin (FB1), an inhibitor of ceramide synthase [33], abrogated the restorative effect of PDMP on p53 expression, thus decreasing the levels of pp53 (50-fold) and increasing β-catenin expression (2-fold) as well as Zeb1 (7-fold) in TP53-Dox cells treated with PDMP plus FB1, as compared to PDMP treatment (Figure 8A, 8B). Silencing of p53 expression with siRNA-p53 [37], which clearly suppresses p53-R273H along with that of wt p53, also decreased pp53 production (>50-fold), increased β-catenin (2-fold) and Zeb1 protein levels (6-fold) (Figures 8A, 8B), and accordingly increased the IC_50_ of doxorubicin (Figures 8C, 8D) in TP53-Dox cells treated with siRNA-p53, as compared to siRNA-SC or PDMP treatment. These results indicate that substantive restoration of wt p53 expression and function, mediated by ceramide, sensitized drug-resistant R273H cancer cells to doxorubicin. Further, ceramide-modulated wt p53 activity in TP53-Dox cells treated with PDMP or MBO-asGCS significantly decreased the protein levels of Zeb1 and β-catenin (Figure 8B), which are essential factors involved in EMT, as noted above. Taken together, these results indicate that PDMP increases cellular ceramide levels as mechanism for restoring wt p53 tumor suppression activity in cancer cells heterozygously carrying p53 R273H mutation, as sketched in Figure 9.

## DISCUSSION

We demonstrated that the presence of heterozygous p53 R273H mutant is an underlying factor promoting the occurrence of iPSCs in colon cancer cells, and in xenografted tumors exposed to chemotherapeutic agents. GOF associated with mutant p53 proteins can enhance the ability of cancer cells to invade, metastasize, and poorly respond to chemotherapies, all of which attributes to CSC presence and pluripotent stem-like phenotypic character [38–40]. p53 clearly plays a major role in iPSC generation from noncancerous cells, in both attenuating reprogramming and controlling the qualities of reprogrammed cells [41]. p53 function in iPSCs exerts an indirect effect on proliferation arrest and on the restriction of mesenchymal-epithelial transition (MET) during its early phases [42]. Inactivation of p53 function is associated with the acquisition of stem-like phenotypic character in reprogrammed cells and in cancers [14, 43–46]. Heterozygous p53 missense mutations are more common than any other mutants in cancers. This is particularly true for sarcomas of Li-Fraumeni syndrome patients. Patients with p53 mutant cancers often have poor prognoses in treatments; however, to date it remains unclear which particular p53 mutants cause this to be so, how significant the impacts are for each mutant, and in each case the exact mechanistic bases for the clinical observations with respect to chemotherapy resistance. The studies presented herein, carried out in cell culture and in xenografted tumor-bearing mice, showed that low-dose doxorubicin induced EMT (Figure 1), augmented CSC numbers, promoted tumor growth, and conferred drug resistance (Figures 1, 3, 5, 7, 8) for cancer cells carrying a heterozygous p53 R273H mutation.

R273 is a mutation hotspot codon, with variants R273H, R273C and R273G occurring most commonly in tumor samples from patients. R273H and R273C, for which expression levels do rise in response to DNA damage and repair, bind less tightly to DNA, and tend to stimulate production of proteins having protective and repair functions, including BRCA1, TOPBP1 and MDC1, thereby leading to a more-aggressive phenotype [47]. We found that the presence of the heterozygous R273H allele led to inadequate levels (or even complete lack of appearance) of the p53-responsive proteins p21, Bax and Puma, which are normally induced in response to p53 after DNA damage. The response failure is especially noteworthy for p21, which strongly constrains cell proliferation in normally differentiated, non-cancerous cells [13]. More importantly, in the R273H mutant-harboring cells, upregulated expression of Zeb1, β-catenin, TGF-β, c-Myc, and Oct4 was observed; these transcription factors reportedly can de-differentiate adult cells [12] and augment the malignant potential of reprogrammed cells after transduction [14, 48]. Furthermore, the heterozygous presence of R273H mutant alone did not intrinsically confer drug resistance or directly induce EMT in cultured SW48/TP53 cells (Figures 1, 8); these characteristics arose only following chronic low-dose doxorubicin exposure, and extended to other chemotherapeutic agents besides doxorubicin. These observations indicate that the presence of the p53 missense mutation acts as an underlying promoting factor for the generation of iPSCs in tumors exposed chronically to anticancer drugs.

Resuscitating normal function of the p53 mutant protein when its mutant forms are extant constitutes an attractive therapeutic strategy for cancer treatments [49], and restoration of wt p53 protein expression levels and functional normalcy holds promise for targeting the majority of p53-mutant cancers more effectively [50]. The tumor-suppressing functions of p53 mainly rely on binding of its homotetrameric form to DNA, thereupon suitably activating or repressing the expression of p53-responsive genes. Restoration of wt p53 expression can switch mutant phenotype to wt by reducing the formation of heterotetramers (wt proteins with mutant proteins) that bind to DNA in place of normal homotetramer, bringing about oncogenic GOF in p53-mutant-harboring cancer cells [50].

Our previous studies showed that inhibition of ceramide glycosylation, either by silencing of GCS expression with MBO-asGCS or by inhibiting GCS activity with PDMP, increased cellular ceramide levels and restored wt p53 expression as well as p53-dependent apoptosis in cancer cells carrying p53 deletion mutants [32, 33]. In the study we report herein, suppression of ceramide glycosylation restored wt p53 expression in TP53 cells that carry a R273H missense mutation allele, with dramatic increases in the protein levels of pp53 and p21 (Figures 4A, 6A). As a downstream consequence, restoration of p53 function further prevented GOF-associated induced pluripotency of CSCs (Figures 1B, 3, 5, 7A), by bring about decreased expression of Zeb1, β-catenin, TGF-β, cMyc and Oct4 (Figures 4, 7). Previous studies showed that targeting GCS overexpression enhanced the sensitivity of cancer cells to anticancer drugs and decreased tumor formation [36, 51–55]. In drug-resistant cancer cells, elevated ceramide glycosylation led to increased levels of globotriaosylceramide (Gb3), which can upregulate multidrug resistant gene 1 (MDR1) [52, 53] through activation of the β-catenin signaling pathway. Increased levels of Gb3 and gangliosides GD2 caused enrichment with CSCs in breast cancers; accordingly, inhibition of GCS lowered CSC numbers, as well as tumor formation [54, 55]. The novel finding in present study is that the increased levels of ceramide (Figures 2B, 2C, 8A, 8B), rather than decreased levels of glycosphingolipids, eliminated oncogenic GOF of p53 in iPSC, via reactivation of wt p53 expression in cancer cells carrying a heterozygous p53 missense mutation.

To our knowledge, this might be the first study showing that normal p53 expression and function can be restored in cancer cells heterozygously harboring a p53 missense mutant allele, in this case a R273H. A recent study documented that activation of chaperone-mediated autophagy degrades mutant p53, including R175H and R273H, and sensitizes cancer cells to treatment-responsive death [56]. Ceramide can mediate phosphorylation of SRSF1 to select wt p53 mRNA for protein expression in cancer cells carrying p53 deletion mutation [33]. Yu *et al.* recently reported that NSC319726, a thiosemicarbazone compound, can reactivate an R175 p53 mutant so as to upregulate the expression of p21, and thereby induce apoptosis for cancer treatment [57]. All these indicate it is feasible to reactivate wt p53 expression and anticancer activity in cancer cells carrying missense mutation, although further studies are required to figure out how post-transcriptional processing modulates p53 expression.

## MATERIALS AND METHODS

### Cell culture and treatments

Human COLO 320DM (homozygous R248W p53) and WiDr (homozygous R273H p53) colon cancer cell lines, and MCF-12A (wild-type p53) immortalized epithelial cells were purchased from ATCC (Manassas, VA). COLO 320DM and WiDr were cultured in RPMI-1640 or ATCC-formulated EMEM containing 10% fetal bovine serum (FBS), 100 units/mL penicillin, 100 μg/mL streptomycin and 584 mg/L l-glutamine. MCF-12A cells were cultured in Dulbecco's modified Eagle's medium-F12 (1:1) with 5% horse serum, insulin (5 μg/ml), hydrocortisone (500 ng/ml), human epidermal growth factor (20 ng/ml) and cholera toxin (100 ng/ml). Human colon cancer SW48 cells and also cells of its corresponding TP53 missense mutant (R273H/^+^) line, were purchased from Horizon Discovery (HD 103-008, Waterbeach, Cambridge, UK) [28]. SW48 cells were cultured in RPMI-1640 medium containing 10% FBS, 100 units/mL penicillin, 100 mg/mL streptomycin, and 2 mM l-glutamine. SW48/TP53 (TP53) cells were cultured in RPMI 1640 medium including 2 mM l-glutamine and 25 mM sodium bicarbonate supplement with 10% FBS and 800 μg/mL geneticin (G418). Cells were maintained in an incubator humidified with 95% air and 5% CO_2_ at 37°C. SW48-Dox and TP53-Dox, which are sublines of SW48 and SW48/TP53 cells, were cultured in 10% FBS RPMI-1640 medium containing 25 nM doxorubicin (Dox) for 16 weeks (∼26 passages).

### Cell viability assay

Cell viability was determined by quantitation of ATP, an indicator of live cells, using the CellTiter-Glo luminescent cell viability assay (Promega, Madison, WI) kit, as described previously [32, 58]. Briefly, cells (4000 cells/well) were grown in 96-well plates with 10% FBS supplemented RPMI-1640 medium. Cells were treated with test agents in 5% FBS medium for 72 hours. Cell viability was determined by the measurement of ATP in a Synergy HT microplate reader (BioTek, Winnooski, VT, USA), following incubation with CellTiter-Glo reagent. For combination treatment, cells (3×10^6^ /100-mm dish; 4000 cells/well in 96-well plates) were grown in 10% FBS RPMI-1640 medium overnight and then cultured in 5% FBS medium containing *d-threo*-1-phenyl-2-decanoylamino-3-morpholino-1-propanol HCl (PDMP; 5 μM) or fumonisin B1 (FB1, 25 μM) and doxorubicin (Dox; 5∼2.0 μM) for 48 hr. PDMP was purchased from Matreya (State College, PA) and fumonisin B1 (FB1) from Sigma-Aldrich (St. Louis, MO).

### Tumor sphere formation assay

A tumor sphere formation assay was carried out as described previously, with minor modification [54, 59]. Briefly, cells of SW48-Dox and TP53-Dox lines (10 to 10,000 cells/well) were plated in ultralow-attachment 24-well plates (Corning, Lowell, MA) with DMEM-F12 (1:1) medium containing insulin (5 μg/mL), human basic fibroblast growth factor (10 ng/mL), human epidermal growth factor (20 ng/mL) and 0.4% bovine serum albumin (BSA). For PDMP treatments, cells were pretreated with PDMP (5 μM) in 10% FBS RPMI-1640 medium for 6 days; the medium was refreshed on day 4. The cells of spheres (>50 μm) were counted using a hemocytometer following trypsinization. Sphere images (200× magnification) were captured using the EVOS FL cell imaging system with color CCD camera (Life Technologies, Grand Island, NY).

### Gene silencing of GCS and p53

Silencing of GCS and p53 was accomplished as described previously [32, 33, 36]. Mixed-backbone oligonucleotide (MBO-asGCS, 100 nM) or siRNA (siRNA-p53, siRNA-SC; 100 nM) were introduced into TP53-Dox cells (3 × 10^6^ /100-mm dish; 4000 cells/well in 96-well plates) after overnight growth, facilitating with Lipofectamine 2000 in Opti-MEM reduced-serum medium (Invitrogen) for 4 hr. The cells continuously grew in 5% FBS medium for an additional 48 hr. In combination groups, cells were treated with PDMP (5 μM) in Opti-MEM medium for 4 hr, after siRNA-p53 transfection, and further grown in 5% FBS medium. To co-silence GCS and p53, cells were transfected with both MBO-asGCS and siRNA-p53 (or siRNA-SC as a control) simultaneously. In the last 48 hr of treatments, cells were cultured in medium containing 20 nM Dox to induce DNA damage. MBO-asGCS [36] was purchased from Integrated DNA Technologies (Coralville, IA). siRNA targeting human p53 (sc-29435) [37] and the scrambled control (siRNA-SC, sc-37007) were purchased from Santa Cruz Biotechnology (Santa Cruz, CA).

### Western blot analysis

Western blotting was carried out as described previously [33, 54]. Briefly, cells or tissue homogenates were lysed in NP40 cell lysis buffer (Biosource, Camarillo, CA, USA) to extract total cellular proteins once the treatment was finished. Protein was measured by using a bicinchoninic acid (BCA) protein assay kit (Pierce, Rockford, IL, USA). Equal amounts of proteins (50 μg/lane) were resolved by using 4-20% gradient SDS-PAGE (Life Technology). After transferring, blots of nitrocellulose membrane were blocked in 5% fat-free milk in 0.05% Tween-20, 20 mM phosphate-buffered saline, pH 7.4 (PBST), and then incubated with each one of the primary antibodies (1:500 or 1:2000 dilution), at 4°C overnight. After PBS washing, these blots were incubated with corresponding horseradish peroxidase–conjugated secondary antibodies (1:5000 dilutions) and developed using SuperSignal West Femto substrate (Thermo Fisher Scientific). Glyceraldehyde-3-phosphate dehydrogenase (GAPDH) was used as a loading control for cellular protein. Relative protein levels present were calculated from the OD values, normalized against those for GAPDH. Antibodies against human p53 phosphorylated at Ser15, and against c-Myc, Klf4, Zeb1, and Oct4, were purchased from Cell Signaling Technology (Danvers, MA). Antibody for glucosylceramide synthase (GCS) was purchased from GeneScript (Piscataway, NJ). Antibodies for Puma, p21Waf1/Cip1, Bax, p53, E-cadherin, vimentin, TGF-β, β-catenin, and GAPDH were obtained from Santa Cruz Biotechnology (Dallas, TX).

### Immunocytochemistry

Cells (20,000 cells/chamber) were grown in 4-chamber slides for 48 hr. After methanol fixation, cells were blocked with 5% goat serum in phosphate-buffered saline (PBS), and incubated with antibodies against E-cadherin or vimentin, phosphorylated p53 Ser15, ceramide (clone MID 15B4 from Sigma) and p21 (1:100 dilutions) in blocking solution at 4°C, overnight. Primary antibodies were further recognized by Alexa-Fluor 555- or 488-conjugated goat IgG (1:2000), respectively. Cell nuclei were counterstained with DAPI (4′,6-diamidino-2-phenylindole) in mounting solution (Vector Laboratories). Images (200× magnification) were captured using the EVOS FL cell imaging system with color CCD camera (Life Technologies, Grand Island, NY).

Tumors were fixed and maintained in paraffin blocks. Microsections (5 μm) of tumors were stained with hematoxylin and eosin (H&E), and characterized by a pathologist. For immunostaining, antigens were retrieved in steaming sodium citrate buffer (10 mM, 0.05% Tween-20, pH 6.0). After blocking with 5% goat serum in PBST, slides were immunostained, as described above.

### Flow cytometry assay

Flow cytometry was carried out as described previously [21, 54]. For analysis of CSCs, cells of SW48-Dox and TP53-Dox lines were treated with PDMP (5 μM) for 6 days, and then harvested with trypsinization and centrifugation. Suspended cells (10^6^ cells/ml) were incubated with CD44v6 Alexa-Fluor 488-conjugated antibody (2F10; mouse IgG1; purchased from R&D Systems, Minneapolis, MN) and CD133/2 APC-conjugated antibody (293C3, mouse IgG2b; purchased from Miltenyi Biotec, San Diego, CA) in 1% BSA-containing PBS at 4°C for 45 min. After washing, cells were resuspended in 1% BSA PBS (1 mL) and analyzed by flow cytometry, using a FACSCalibur instrument (BD Biosciences, San Jose, CA) operated with CellQuest Pro software (BD Biosciences), and the data were further analyzed by using the FlowJo program (v10; FlowJo, Ashland, OR). For each sample, 10,000 events were counted in triplicate. To identify CD44v6^+^/CD133^+^ cells, samples obtained under different treatments were incubated in 1% BSA PBS for measuring autofluorescence.

To analyze CSCs in mice, resected tumors (∼60 mg) were immediately dispersed in RPMI-1640 medium with collagenase IV (500 units/mL) at 37°C for 120 min with shaking (20 rpm). After filtration through a 70-μm cell strainer, cells were incubated with APC-CD44 v6 and PE-CD133/2 IgG for flow cytometry analysis as described above. Collected bone marrow cells (BMCs) from each mouse were counted with a hemocytometer. The ABCG2^+^ BMCs were analyzed by flow cytometry, following the incubation of BMCs with anti-ABCG2 antibody, as described previously [21].

### Glucosylceramide synthase assay

GCS activity was assayed as described previously [60, 61], with modification. Briefly, cells were grown 24 hr in 35-mm dishes (5×10^6^ cells/dish) in 10% FBS RPMI-1640 medium, and then switched to 1% BSA RPMI-1640 medium containing 2.0 μM 7-nitro-2,1,3-benzoxadiazole (NBD) C6-ceramide complexed to BSA (Invitrogen). After 2 h incubation at 37°C, cellular lipids were extracted and reconstituted with chloroform/methanol (1:1, v/v; 200 fluorescence units/100 μl). From this preparation, a 5-μl of portion was loaded by autosampler onto a normal-phase silica column (ZORBAX Rx-SIL, 5 μm, 4.6 × 250 mm). NBD sphingolipids were eluted by linear gradient (0-14 min, 1 ml/min) using solvent system A (chloroform/methanol/*ortho*-phosphoric acid) (80:20:0.1, v/v/v) and solvent system B (chloroform/methanol/H_2_O/*ortho*-phosphoric acid) (60:34:6:0.1, v/v/v/v), and detected with an Agilent 1260 fluorescence detector (λ_excitation_= 470 nm, λ_emission_ = 530 nm) (Agilent, Santa Clara, CA; HPLC system: Agilent 1220 Infinity LC Gradient System VL). Individual sphingolipids were identified and quantitated with a separate standard curve for each. Each sample was analyzed at least three times, and GCS activity is defined in terms of glucosylceramide produced in 2 hr normalized against total cellular proteins. To assess tumor GCS activity, tumors were pieced (<1 mm) and digested with collagenase IV for 2 hr, and samples were then prepared and processed as described above.

### ESI/MS/MS analysis of ceramides

Speciation of endogenous sphingolipids was accomplished with a Thermo-Fisher TSQ Quantum triple quadrupole mass spectrometer, operated in a Multiple Reaction Monitoring (MRM) positive ionization mode, as described previously [33, 62, 63]. Total cells, fortified with internal standards, were extracted with ethyl acetate/isopropanol/water (60/30/10 v/v). These extracts were evaporated to dryness, and reconstituted in 100 μl of methanol. Portions of the reconstituted samples were injected on the Surveyor/TSQ Quantum LC/MS system, and gradient-eluted from the BDS Hypersil C8 column (150 × 3.2 mm, 3 μm) with a 1.0 mM methanolic ammonium formate/2 mM aqueous ammonium formated mobile phase system. The peaks for the target analytes and internal standards were identified and processed using the Xcalibur software. Calibration curves were constructed by plotting peak area ratios of synthetic standards, representing each target analyte, to the corresponding internal standard. Concentrations for samples were obtained from these calibration curves by linear regression, and cellular sphingolipids levels calculated by normalization against total cellular protein.

### Tumor-bearing mice and treatments

All animal experiments were approved by the Institutional Animal Care and Use Committee, University of Louisiana at Monroe (ULM), and were carried out in strict accordance with good animal practice as defined by NIH guidelines. Athymic nude mice (Foxn1^nu^/Foxn1^+^, 4-5 weeks, female) were purchased from Harlan (Indianapolis, IN) and maintained in the vivarium at ULM. Animal studies were conducted as described previously [32, 36, 53]. Briefly, a cell suspension of SW48 and SW48/TP53 (2-3 passages, 1×10^6^ cells in 20 μl/mouse) was subcutaneously injected in the left flank of the mice. Mice were monitored by measuring tumor growth and body weight, under clinical observation. Once tumors were visible (2 mm in diameter), mice were randomly allotted to different treatment groups (5 mice/group). For treatments, PDMP (4.0 mg/kg once every 3 days) was administered intraperitoneally alone or with doxorubicin (200 or 300 μg/kg once every 6 days) for 32 days. Tumor volume was approximated by the formula L/2 × W^2^ (where L is the length and W is the width). Tumors and metastases were examined and characterized by pathologist following H&E staining of tissue sections at Louisiana State University Health Sciences Center (Shreveport, LA).

### Data analysis

All experiments were repeated 2 or 3 times. Data are expressed as mean ± SD. Two-tailed Student's *t* tests and ANOVA tests were used to compare the continuous variables in groups, using the Prism v5 program (GraphPad, San Diego, CA). All *p*<0.05 comparisons were regarded as statistically significant.

## SUPPLEMENTARY FIGURE


