# Supplementary material for: Inhibition of glucosylceramide synthase eliminates the oncogenic function of p53 R273H mutant in the epithelial-mesenchymal transition and induced pluripotency of colon cancer cells
Source: Oncotarget. 2016 Aug 10;7(37):60575–92. doi: 10.18632/oncotarget.11169 (PMC5312403; doi:10.18632/oncotarget.11169)
Supplement: Supplementary file 1 [file oncotarget-07-60575-s001.pdf]

Inhibition of glucosylceramide synthase eliminates the oncogenic function of p53 R273H mutant in the epithelial-mesenchymal transition and induced pluripotency of colon cancer cells

SUPPLEMENTARY FIGURE

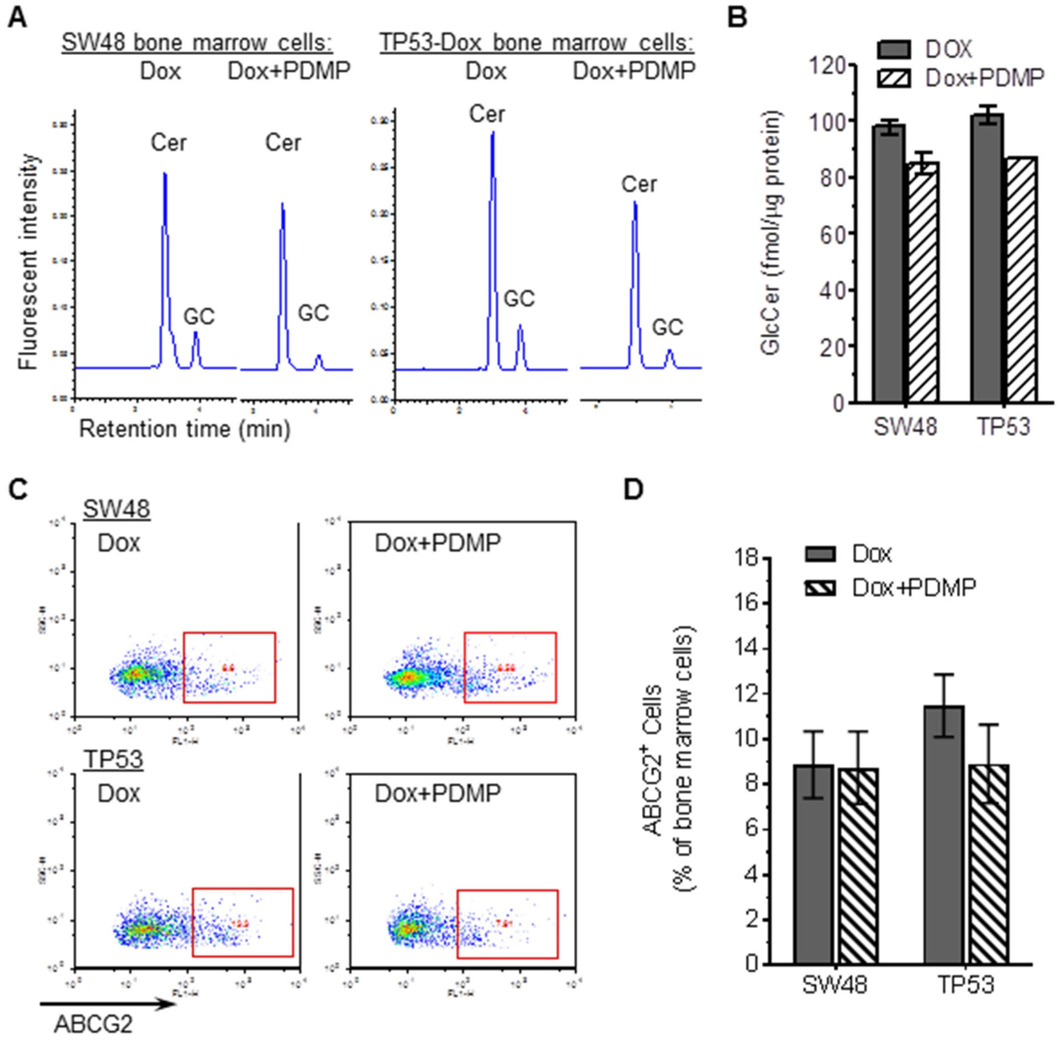

Supplementary Figure S1: Effects of PDMP and Doxorubicin Treatments on Mouse Bone Marrow. After 32 days of the treatments (Dox, 200 μg/kg, i.p. per 6 days; Dox+PDMP, 5 μg/kg, i.p., per 3 days), bone marrow cells were extracted and used for analyses. **A, B.** GCS activities assessed by HPLC analysis. **C, D.** Bone marrow stem cells (ABCG2^{+}) assessed by flow cytometry.
